# First insight into the whole-genome sequence variations in *Mycobacterium bovis* BCG-1 (Russia) vaccine seed lots and their progeny clinical isolates from children with BCG-induced adverse events

**DOI:** 10.1186/s12864-020-06973-5

**Published:** 2020-08-18

**Authors:** Olga Narvskaya, Daria Starkova, Diana Levi, Natalia Alexandrova, Vladimir Molchanov, Ekaterina Chernyaeva, Anna Vyazovaya, Alexander Mushkin, Viacheslav Zhuravlev, Natalia Solovieva, Boris Vishnevskiy, Igor Mokrousov

**Affiliations:** 1 Laboratory of Molecular Epidemiology and Evolutionary Genetics, St. Petersburg Pasteur Institute, St. Petersburg, 197101 Russia; 2 St. Petersburg Research Institute of Phthisiopulmonology, St. Petersburg, 191036 Russia; 3 Scientific Center for Expert Evaluation of Medical Products, Moscow, 127051 Russia; 4 Present address: Van Andel Institute, Grand Rapids, MI 49503-2518 USA; 5 Saint Petersburg State University, St. Petersburg, 199034 Russia

**Keywords:** BCG, *M. bovis* BCG-1 (Russia), Seed lot, Clinical isolate, Osteitis, Whole-genome sequencing

## Abstract

**Background:**

The only licensed live Bacille Calmette-Guérin (BCG) vaccine used to prevent severe childhood tuberculosis comprises genetically divergent strains with variable protective efficacy and rates of BCG-induced adverse events. The whole-genome sequencing (WGS) allowed evaluating the genome stability of BCG strains and the impact of spontaneous heterogeneity in seed and commercial lots on the efficacy of BCG-vaccines in different countries. Our study aimed to assess sequence variations and their putative effects on genes and protein functions in the BCG-1 (Russia) seed lots compared to their progeny isolates available from immunocompetent children with BCG-induced disease (mainly, osteitis).

**Results:**

Based on the WGS data, we analyzed the links between seed lots 361, 367, and 368 used for vaccine manufacture in Russia in different periods, and their nine progeny isolates recovered from immunocompetent children with BCG-induced disease. The complete catalog of variants in genes relative to the reference genome (GenBank: CP013741) included 4 synonymous and 8 nonsynonymous single nucleotide polymorphisms, and 3 frameshift deletions. Seed lot 361 shared variants with 2 of 6 descendant isolates that had higher proportions of such polymorphisms in several genes, including *ppsC*, *eccD5,* and *eccA5* involved in metabolism and cell wall processes and reportedly associated with virulence in mycobacteria. One isolate preserved variants of its parent seed lot 361 without gain of further changes in the sequence profile within 14 years.

**Conclusions:**

The background genomic information allowed us for the first time to follow the BCG diversity starting from the freeze-dried seed lots to descendant clinical isolates. Sequence variations in several genes of seed lot 361 did not alter the genomic stability and viability of the vaccine and appeared accumulated in isolates during the survival in the human organism. The impact of the observed variations in the context of association with the development of BCG-induced disease should be evaluated in parallel with the immune status and host genetics. Comparative genomic studies of BCG seed lots and their descendant clinical isolates represent a beneficial approach to better understand the molecular bases of efficacy and adverse events during the long-term survival of BCG in the host organism.

## Background

Treatment of tuberculosis (TB) and vaccination remain inevitable health-care interventions to prevent new *Mycobacterium tuberculosis* infections and their progression to disease. The live Bacille Calmette-Guérin (BCG) vaccine is the only licensed vaccine that is successfully used for nearly a century to prevent childhood TB, although it shows a modest protective effect in adults [1, 2]. The history of BCG development and spreading was thoroughly reviewed [3, 4]. Briefly, the ancestral form of BCG was derived from wild-type *Mycobacterium bovis* progenitor strain, while attenuation by numerous in vitro passages resulted in world distribution of parental BCG strain in 1924. During the next decades, maintenance of the BCG strain progenies by further passages using different culture media and transfer schedules in different countries contributed to the in vitro evolution of BCG. The production of seed stocks without lyophilization until the 1960s resulted in the generation of BCG daughter strains (sub-strains) with variable morphological, culture, antigenic characteristics, and residual virulence due to adaptation in vitro [3–7].

The up-to-date genealogy of widely used BCG vaccine strains is based on genomic variations that occurred due to large deletions, tandem duplications, insertion sequences, and the subsequent series of insertions/deletions and single nucleotide polymorphisms (SNPs). Accordingly, BCG strains were classified into “early” strains represented by BCG Japan, Moreau, Russia (and the descendant BCG Sofia) strains that show fewer chromosomal deletions than “late” strains - Danish, Pasteur, Glaxo, and others [8–11]. Altogether, these variations underpin the molecular basis of attenuation, different levels of gene expression of immunodominant proteins leading to variable protective efficacy, and different rates of adverse events of currently used genetically divergent BCG strains otherwise considered different BCG vaccines [3, 8].

Historically, the original *M. bovis* BCG strain was transferred to Russia in 1924, and since 1925 it was referred to as *M. bovis* BCG-1 (also spelled as BCG-I). In the 1940s, *M. bovis* BCG-1 was lyophilized, and in 1954 the seed lot system was adopted in Russia. The first seed lot used in 1963 for the manufacturing of freeze-dried BCG vaccines in the former Soviet Union was seed lot 352 (“ch”). Since 2006, the 368 (“shch”) generation of *M. bovis* BCG-1 (Russia) strain no. 700001 is in use for the production of the BCG vaccine in Russia [12, 13]. The same BCG generation 368 is also adopted by two manufacturers in India for vaccine production and distribution worldwide under the name “BCG Russia 368” [14].

The attenuated BCG vaccines are believed safe and effective mainly to prevent the most severe meningitis and miliary forms of childhood TB associated with high mortality in infants and young children, especially in high TB-burden countries [1, 2]. However, adverse events following immunization with BCG in 1–10% of vaccinees are likely to be substantially underreported since assays to confirm isolates as BCG are still relatively rare [15–20] despite the advent of molecular techniques and commercially available kits for BCG identification [21]. In Russia, the estimated frequency of complications per 100,000 BCG-immunized decreased from 35.3 in 2005 to 11.2 in 2013 [22].

Vaccine-related complications, i.e., BCG-induced disease (BCG-ID), may occur later than 12 months after vaccination as local site reactions or distal to the site of inoculation, and they appear to differ from cutaneous lesions, lymphadenopathy/lymphadenitis, and hypersensitivity reactions to osteitis and disseminated BCG infections (almost exclusively in individuals with compromised cellular immunity) [1, 16, 19, 23–25].

The BCG-induced adverse reactions are believed to be due to the use of genetically distant vaccine strains with variable residual virulence, the culture technique, the route of administration and the dose of viable cells in the BCG vaccine delivered, along with the age, immune status, and host genetics of the vaccinees [4, 8, 9, 16, 17, 26, 27].

To standardize the vaccine production and to prevent adverse reactions related to BCG vaccination, the WHO has established the international requirements for the manufacture and quality control including genetic characterization of seeds and final commercial lots of the reference strains, i.e., BCG Danish 1331, Tokyo 172–1, and Russia BCG-1 used for vaccine manufacture and distribution worldwide [11, 28, 29].

Given recommendations, the identity and genome stability of seven seed lots of *M. bovis* BCG-1 (Russia) used for immunization against TB in Russia since 1948 till present, and 32 commercial lots of the vaccine produced by Russian manufacturers were proved by multiplex polymerase chain reaction [12].

During the last decades, the whole-genome sequencing (WGS) was applied to assess the genome stability of BCG vaccine strains from different culture collections and to evaluate the impact of minor sequence variations occurring in seed and commercial lots on the efficacy of BCG-vaccines used in different countries [30–34]. Recently, sequence variations were identified in clinical isolates from two infants with BCG-induced disease compared with the sub-cultured *M. bovis* BCG Moscow strain (Serum Institute of India, India) in South Africa [15].

The complete genome sequences of the current *M. bovis* BCG-1 (Russia) 368 (“shch”) freeze-dried seed lot and its working seed lot used for vaccine production by one of the Russian manufacturers are available in GenBank (NCBI) under accession numbers CP011455 [13], CP009243 [35], and CP013741 [36]. The genome constancy of *M. bovis* BCG-1 (Russia) 368 within the entire vaccine production flow (from working seed lot, the last production passage to final vaccine product) was confirmed by spoligotyping, 24 loci VNTR-typing, and WGS [36, 37]. However, so far, there is a lack of data on the contribution of BCG genomic diversity to the viability, fitness, and residual virulence of vaccine populations in vivo in the context of the development of BCG-induced adverse events. Therefore, our study aimed to assess sequence variations and their putative effects on genes and protein functions in the BCG-1 (Russia) seed lots compared to their progeny clinical isolates available from immunocompetent children with BCG-induced osteitis and lymphadenitis.

## Results

### Sequence variations identified in BCG-1 (Russia) parent seed lots and their progeny clinical isolates

Raw reads of seed lots 361, 367, 368, and descendant clinical isolates were mapped to the reference genome of BCG-1 (Russia) (GenBank: CP013741). Variant calling allowed to produce the complete catalog of 15 differences, including 12 SNPs (four synonymous and eight nonsynonymous) and three disruptive deletions (frameshift variants) in CDS of the seed lots and clinical isolates relative to the reference genome. Overview of sequence variation patterns identified in BCG seed lots and their progeny clinical isolates are presented in Table 1, Additional Files 1 and 2.

**Table 1:** Sequence variants in CDS of BCG-1 (Russia) parent seed lots 361, 367 and 368 and their progeny clinical isolates compared to the reference sequence BCG-1 (Russia) (GenBank: CP013741)

Parent gene BOVR	Parent gene BOVR codon change	Chain	Seed lot 361	1986	2925	4159	5075	5340	5448	Seed lot 367	3363	Seed lot 368	577	1032	Parent gene BOVR amino acid change	Product name	Effect (putative impact)
01860	509delC	–	CG	CG	CG	CG	CG	CG	CG	CG	C 1.0	CG	CG	CG	Pro170fs	thioesterase	high
03020	121C > T	–	C	C	C	C	C	T 0.37	C	C	C	C	C	C	Ala41Thr	PE-PGRS family protein PE_PGRS7	moderate
03865	685delA	+	CA	CA	CA	CA	CA	CA	CA	CA	CA	C 0.69	C 0.47	CA	Thr229fs	methionine aminopeptidase	high
06950	1403 T > C	+	T	T	T	T	T	T	T	T	T	T	C 0.43	T	Leu468Ser	ATP synthase subunit beta	moderate
08645	2004A > G	+	G 0.42	A	G 1.0	A	A	A	A	A	A	A	A	A	Val668Val	polyketide synthase	low
08855	1115A > G	–	G 0.18	A	A	A	A	A	G 1.0	A	A	A	A	A	Leu372Pro	amino acid transporter	moderate
09330	982G > A	+	A 0.40	G	A 0.96	G	G	G	A 1.0	G	G	G	G	G	Ala328Thr	type VII secretion integral membrane protein EccD	moderate
09345	128 T > C	+	C 0.33	T	C 1.0	T	T	T	C 1.0	T	T	T	T	T	Ile43Thr	type VII secretion AAA-ATPase EccA	moderate
10,325	634delC	+	GC	GC	GC	GC	GC	GC	GC	GC	GC	GC	GC	G 0.28	Leu212fs	universal stress protein UspA	high
10,615	587C > T	–	T 0.18	C	T 1.0	C	C	C	T 1.0	C	C	C	C	C	Gly196Asp	dolichol-phosphate mannosyltransferase	moderate
12,050	78C > A	–	A 0.29	C	C	C	C	C	C	C	C	C	C	C	Leu26Leu	ornithine aminotransferase	low
13,820-	755C > T	+	T 0.54	C	T 1.0	C	C	C	T 0.89	C	C	C	C	C	Ala252Val	protease	moderate
15,090	2139A > G	–	G 1.0	G 1.0	G 1.0	G 1.0	G 1.0	G 1.0	G 1.0	G 1.0	G 1.0	G 0.25	G 0.46	G 1.0	Ala713Ala	protein-PII uridylyltransferase	low
16,925	846C > T	–	C	C	C	C	C	C	C	C	C	C	C	T 0.18	Leu282Leu	N-acetylglucosaminyl-diphospho-decaprenol L-rhamnosyltransferase	low
17,690	833A > G	+	A	A	A	A	A	A	A	A	G 1.0	A	A	A	Gln278Arg	amidase	moderate

Sequence variants are presented as the proportion of reads with alternative allele/all reads; *del* deletion, *fs* frameshift

His57Asp mutation in *pncA* (Rv2043c) gene conferring high intrinsic resistance to pyrazinamide in *M. bovis* was detected in all BCG-1 (Russia) progeny isolates. A single-nucleotide insertion in the *recA* gene was not detected in any of three seed lots, nor their descendant clinical isolates.

### Comparative analysis of sequence variants in BCG-1 (Russia) seed lots and their progeny clinical isolates

We examined a set of nine BCG-1 (Russia) clinical isolates recovered from duly BCG-vaccinated immunocompetent children (except one, immunized with delay for 4 months after birth) with a culture-confirmed diagnosis of BCG-ID. For each case, analysis of medical records allowed to follow the history of primary BCG vaccination, including information on commercial vaccine products used for intradermal inoculation, its manufacture, and finally, the parent seed lot (Tables 2 and 3).

**Table 2:** Characteristics of patients and *M. bovis* BCG-1 (Russia) clinical isolates

Patient ID/sex^a^	Date of birth^b^	Region of birth	Vaccination	Vaccine lot number used for inoculation	Vaccine manufacturer	Vaccine seed lot	Diagnosis^c^	Age of surgical intervention^d^	Date of surgical intervention^h^	Clinical isolate	NCBI SRA accession No
1/f	05/2017	St. Petersburg	delayed ^e^	411	Medgamal ^f^	368 “shch”	A18.2	7 m	01/2018	577	SRR9593177
2/m	06/2016	Dagestan	at birth	537	Microgen ^g^	368 “shch”	A18.0	1y8m	02/2018	1032	SRR9593172
3/m	07/2006	Moscow	at birth	621	Medgamal	361 “sh”	A18.0	9 m	04/2007	1986	SRR9593171
4/f	03/2002	St. Petersburg	at birth	281	Medgamal	361 “sh”	A18.0	14y	04/2016	2925	SRR9593170
5/f	07/2004	Ekaterinburg	at birth	25	Microgen	367 “sh”	A18.0	2y10m	05/2007	3363	SRR9593169
6/f	12/2004	St. Petersburg	at birth	437	Medgamal	361 “sh”	A18.0	2y6m	06/2007	4159	SRR9593168
7/m	04/2006	St. Petersburg	at birth	615	Medgamal	361 “sh”	A18.0	1y5m	09/2007	5075	SRR9593175
8/m	12/2004	St. Petersburg	at birth	725	Medgamal	361 “sh”	A18.0	4y9 m	09/2009	5340	SRR9593174
9/m	06/2005	Kabardino-Balkaria	at birth	567	Medgamal	361 “sh”	A18.0	1y3m	09/2006	5448	SRR9593173

^a^*f* female, *m* male; ^b^month/year; ^c^diagnosis according to International Statistical Classification of Diseases and Related Health Problems (ICD-10): Tuberculosis of bones and joints (A18.0), A18.2 (Tuberculosis peripheral lymphadenopathy) [International statistical classification of diseases and related health problems. - 10th revision, edition 2010]; ^d^*y* year, *m* month; ^e^delayed for 4 months due to respiratory infection; ^f^Branch “Medgamal,” Federal State Budgetary Institution “National Research Center for Epidemiology and Microbiology named after Honorary Academician N.F. Gamalei”, Ministry of Health of the Russian Federation, Russia; ^g^Scientific and Production Association for Immunobiological Preparations “Microgen,” Russia; ^h^month/year

**Table 3:** Seed lots of *M. bovis* BCG-1 (Russia) substrain No. 700001

Freeze-dried seed lot^a^	Date of lyophilization ^b^	Origin	Used for vaccine manufacturing	Manufacture	NCBI SRA accession number
361 “sh”	09/1992	Freeze-dried Seed lot 352 “ch” (lyophilization 1963)	2002–2007	Medgamal	SRR9593176
367 “shch”	11/1982	Freeze-dried Seed lot 359 “sh” (lyophilization 1966)	2001–2008	Microgen	SRR9593167
368 “shch”	08/2006	Freeze-dried Seed lot 359 “sh” (lyophilization 1966)	2008 till present	Medgamal, Microgen	SRR9593166

^a^ the lyophilized seed lots (generations 361 “sh,” 367 “shch,” and 368 “shch”) of *M. bovis* BCG-1 (Russia) substrain No. 700001 in ampoules were obtained from the National State Collection of Pathogenic Microorganisms (NSCPM) of Scientific Center for Expertise of Medical Application Products of the Ministry of Health, Russia along with the characteristics (the origin and manufacture) of corresponding BCG vaccine commercial lots used for immunization; ^b^ month/year

**Fig. 1:**
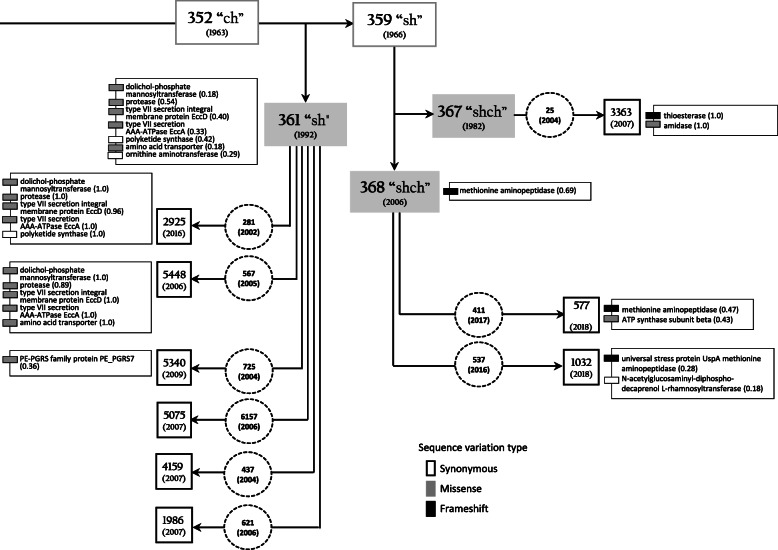
Schematic view of relationships between vaccine strain BCG-1 (Russia) parent seed lots, commercial vaccine lots, and their progeny clinical strains. Rectangle boxes correspond to the original seed lots with dates of lyophilization shown in parentheses. Rectangle shadowed boxes depict the sequenced seed lots (dates of lyophilization shown in parentheses). Dashed circles show commercial vaccine lots (were not available for sequencing) used for immunization with dates of inoculation in parentheses. Square boxes mark sequenced BCG clinical isolates (dates of BCG culture isolation shown in parentheses). Gray-scale small boxes depict sequence variants in genes related to particular proteins (proportions shown in parentheses) in seed lots 361, 367, 368, and their progeny clinical isolates. See Table 1 for more details

BCG clinical isolates shared the majority of genomic features with their parent seed lots. However, the proportions of reads with alternative alleles varied in BCG seed lots, and their progeny isolates. These findings were summarized in Table 1 and Additional file 1. Based on the comparative analyses of the data obtained, we built a schematic view of the relationships between BCG seed lots and their descendant clinical isolates (Fig. 1).

The population of BCG-1 (Russia) seed lot 361 was polymorphic with variable proportions of SNPs and deletions in reads spanning seven CDS (Table 1, Fig. 1, Additional file 1).

Synonymous SNP in the ornithine aminotransferase gene (*rocD*) was detected in almost 1/3 reads of seed lot 361, but it was absent in the descendant isolates. Alternatively, seed lot 361 shared nonsynonymous (missense) SNPs in type VII secretion system ESX-5 (*eccD5* and *eccA5),* dolichol-phosphate mannosyltransferase (*ppm1)* and hypothetical protease genes with its progeny clinical isolates 2925 and 5448 that had higher proportions (up to 100%) of alternative variants. However, sequences of these isolates differed in amino acid transporter (*cycA*) and polyketide synthase (*ppsC*) genes. A nonsynonymous SNP in the PE-PGRS family protein PE_PGRS7 gene was found in 38% reads of isolate 5340, the progeny of seed lot 361.

Isolates 1986, 4159, and 5075, progenies of the seed lot 361, and seed lot 367 did not show sequence polymorphisms compared to the reference sequence, i.e., BCG-1 (Russia) current seed lot 368 (Table 1, Additional file 1).

Isolate 3363, a single progeny of the seed lot 367, carried a missense variant in amidase (*amiD*) gene and a frameshift deletion in the thioesterase (*pks13*) gene (Table 1, Fig. 1, Additional file 1).

The sequenced seed lot 368 shared a single base deletion in methionine aminopeptidase (*mapA*) with descendant isolate 577, also demonstrating a polymorphism (43% reads) in the ATP synthase subunit beta gene (*atpD*). Polymorphism in the N-acetylglucosaminyl-diphospho-decaprenol L-rhamnosyltransferase gene (*wbbL1*) and a frameshift deletion in the universal stress protein UspA gene were identified in clinical isolate 1032 (18 and 28% reads, respectively), the progeny of seed lot 368 (Table 1, Fig. 1; Additional file 1 and 3).

## Discussion

All nine BCG clinical isolates recovered from tissue samples taken during the surgery were susceptible to first-line antibiotics: streptomycin, isoniazid, rifampin, ethambutol. However, they were pyrazinamide-resistant due to the typical His57Asp mutation in *pncA* (Rv2043c) gene conferring high intrinsic resistance in *M. bovis,* and consequently, all BCG vaccine strains [38].

Previously, spontaneous heterogeneity of BCG seed lots and commercial vaccines during vaccine production determined by variant calling was analyzed in the Tokyo-172 vaccine strain [32]. In line with the published data, we observed a synonymous A2139G single nucleotide base substitution (position 3,175,529, “-” chain) in BOVR_15,090, i.e., the *glnD* (ortholog Rv2918c in *M. tuberculosis* H37Rv) gene, without Ala replacement in position 713 of 808 amino acids in the conservative domain of the PII uridylyltransferase protein in all genomes studied. The G variant was present in 100% reads of sequenced seed lots 361 and 367, and eight of nine clinical isolates. However, it was detected only in 25% reads of current seed lot 368 (sequenced in this study), and 46% reads of its progeny isolate 577.

These findings inspired further examination of A/G polymorphisms in the *glnD* in other sequenced BCG genomes.

The G variant was previously reported in the *glnD* in BCG-1 (Russia) current seed lot 368 and some earlier vaccine products [35], though alternative variant A was detected in two other BCG-1 (Russia) 368 complete genomes [13, 36]. The stability of variant A in the *glnD* gene of the BCG-1 (Russia) 368 working seed lot (GenBank: CP013741) and consistent vaccine batches within 2 years was confirmed by WGS [36, 37]. Noteworthy, all three complete genomes of BCG-1 (Russia) were sequenced independently from the same generation 368 (“shch”), which is still in use since 2008 for the BCG vaccine production by two Russian manufactures: Medgamal (Moscow) and Microgen (Stavropol).

Interestingly, allele G was recorded in the genomes of BCG str. Tokyo 172 which represents a well-defined Type I subpopulation of BCG Tokyo-172, the closest to BCG Russia (GenBank: NZ_CUWO01000001) and Russia ATCC 35740, members of presumably monophyletic “early” substrain group of BCG [10, 30, 32]. The G variant was also common in the reference sequences of some other BCG strains, e.g., Pasteur 1173P2, Danish 1331, ATCC 35743 (Tice), Moreau RDJ, used worldwide for vaccine production and research purposes.

Altogether, these data suggest that intrinsic polymorphism in the *glnD* gene of BCG-1 (Russia) strain represented by different seed lots and descendant clinical isolates occurs due to the selection of variants during laboratory and vaccine production manipulations, rather than mutations in patients’ organisms. Otherwise, the observed heterogeneity can be partly explained as a result of processing nucleotide variants among the mapped reads using different bioinformatics tools and quality criteria.

Another interesting finding: a notorious single-nucleotide insertion in the *recA* gene leading to nonfunctional truncated recombinase A reported previously in a vaccine production lot of *M. bovis* BCG Russia (corresponding to ATCC 35740) [39] was not detected in seed lots studied, nor their descendant clinical isolates. De facto, there were no alterations in *recA* in the previously sequenced complete genomes of BCG-1 (Russia) [13, 35, 36] along with other hyperconserved BCG strains [33].

Based on the comparative study of WGS data, we attempted to analyze the links between BCG-1 (Russia) seed lots used for vaccine manufacture and their progeny clinical isolates associated with specific adverse events, e.g., BCG-osteitis and an abscess at the point of injection.

The population of BCG-1 (Russia) seed lot 361 appeared heterogeneous with variable proportions of SNPs and deletions in reads spanning seven CDS. Seed lot 361 shared SNPs in *eccD5, eccA5, ppm1* genes*,* and hypothetical protease gene with its progeny clinical isolates 2925 and 5448. The latter typically had higher proportions (reaching up to 100%) of alternative alleles than the parent seed lot 361, thus suggesting the likely accumulation of such changes under selective pressure in the host organism. The similar SNPs we observed in corresponding genomic positions in *M. tuberculosis* H37Rv, BCG strains Tokyo-172 type I and Pasteur 1173P2. These polymorphisms affected type VII specialized bacterial secretion ESX-5 (integral membrane protein EccD and secretion AAA-ATPase), reportedly associated with virulence in mycobacteria, and dolichol-phosphate mannosyltransferase and protease genes involved in the vital cell processes (protein modification, lipoprotein biosynthesis), intermediary metabolism, and respiration [8, 40, 41]. The data obtained confirmed the close genetic relatedness between seed lot 361 and both progeny isolates 2925 and 5448 that otherwise differed from each other in amino acid transporter (*cycA*) and polyketide synthase (*ppsC*) sequences.

A unique missense variant in the PE-PGRS family protein PE_PGRS7 gene with an unclear function in antigenic variation and interactions of bacteria with the host immune system was found in 38% reads of isolate 5340, the progeny of seed lot 361.

Interestingly, no sequence polymorphisms (except *glnD*) were detected in isolates 1986, 4159, and 5075, progenies of the seed lot 361 (used for BCG manufacture in 2002–2007) compared to the reference sequence, i.e., BCG-1 (Russia) current seed lot 368.

The sequences of seed lot 367 (used for vaccine production in 2001–2008) also did not show variations in 14 of 15 CDS compared to the reference genome and thus appeared the most conservative in our study. However, its single progeny, isolate 3363, carried a missense variant in amidase (*amiD*) gene essential for protein translation and required for mycobacterial survival during infection (virulence factor) and a frameshift deletion in the thioesterase region of polyketide synthase 13 (*pks13*) gene involved in the final assembly of mycolic acids, major and specific lipid components of the envelope essential for the survival of a mycobacterial cell [40, 42].

The population of the sequenced seed lot 368 was almost homogenous containing a single base deletion (69% reads) in methionine aminopeptidase (*mapA*) gene compared to the previously reported complete genomes of BCG-1 (Russia) current-generation 368 [13, 35, 36] and BCG Tokyo 172 type 1 [32]. This frameshift leading to protein truncation was inherited in the descendant isolate 577 (47% reads), also bearing an isolate-specific allele (43% reads) in the ATP synthase subunit beta gene (*atpD*). Both proteins encoded by *mapA* and *atpD* genes are involved in the processes of intermediary metabolism and respiration [8].

A synonymous SNP in the N-acetylglucosaminyl-diphospho-decaprenol L-rhamnosyltransferase gene (*wbbL1*) not altering the enzyme structure and function in the cell wall and cell processes was identified in clinical isolate 1032 (18% reads). Another finding in isolate 1032 (28% reads) was a frameshift due to a single-base cytosine deletion in codon 630 of the universal stress protein UspA gene (orthologue Rv1996 in *M. tuberculosis* H37Rv), one of eight Usp paralogues essential for the intracellular survival of pathogenic mycobacteria in hypoxia, low levels of nitric oxide and carbon monoxide environment. This deletion completely altering 27 amino acids from the position 212 onwards resulted in a gained opal stop codon (TGA). The latter was leading to a premature protein truncation in the residue 239 yielding to the loss of 3 ligand-binding loci in the putative ATP binding motif of the UspA domain 2 [43].

It appears that the accumulation of shared SNPs and unique variations occurred in clinical isolates 577 and 1032 (both progenies of the sequenced seed lot 368), 3363 (progeny of seed lot 367), and 5340 (progeny of seed lot 361) did not affect the vaccine survival in the organisms of vaccinees. Moreover, none of the frameshifts, which presumably could alter essential cell functions, were crucial for the microbial population in vitro, nor in vivo, thus emphasizing the long-term viability of BCG-1 (Russia) strain in the human organism.

Adverse events following immunization with BCG are considered underreported since assays to confirm isolates as BCG are still relatively rare. Moreover, based on the routine laboratory diagnostics and even molecular techniques, it is difficult to associate the clinical BCG isolates with the particular vaccine lot administered [15–20]. Therefore, the contribution of BCG genomic heterogeneity to the viability and residual virulence of vaccine populations in vivo remains unclear in terms of the development of BCG-related disease that otherwise can be associated with immune responses and human genetics.

Unfortunately, the administered commercial vaccines were unavailable for WGS, which limited the comparative analysis of sequence variations through the whole vaccine production chain. Nevertheless, bioinformatics tools provided a possibility to identify sequence variants and their putative effects on genes and protein functions in BCG-1(Russia) seed lots 361 and 368 used for vaccine manufacture in Russia in different periods relative to their progeny clinical isolates from patients with confirmed BCG-related disease and the genomes of internationally recognized BCG reference strains.

BCG osteitis is a rare but severe adverse event that typically presents in children before the age of 5 years, and there are few reports concerning the late onset of the disease in teenagers [17, 19]. In our setting, we analyzed confirmed cases of BCG–osteitis typically developed at the age under 5 years (median 22 months) among immunocompetent children immunized with BCG-1 (Russia) vaccine after, or soon after birth. The latest onset of the osteitis affecting the left eighth rib was diagnosed in the patient aged 14 years who once received as a newborn the BCG vaccine derived from the seed lot 361. The isolate 2925 recovered from tissue samples taken during surgery accumulated four nonsynonymous and one synonymous SNPs assigned to its parent seed lot 361 but did not gain any further sequence variants in the patient’s organism. Noteworthy, such a prolonged period of survival in a human organism of the life BCG vaccine without significant changes in its genome appears remarkable.

## Conclusions

To our knowledge, we reported for the first time the results of a WGS-based comparative analysis of the BCG-1 (Russia) seed lots used for the vaccine production in different periods and their progeny clinical isolates recovered from immunocompetent children with BCG-induced adverse reactions (mainly, BCG osteitis). Our study provided the background genomic information, which allowed us to follow the BCG diversity starting from the freeze-dried seed lots to descendant clinical isolates presenting the results of the long-term survival of vaccine populations in the human organism.

Two of three BCG-1 (Russia) seed lots were heterogeneous with various proportions of sequence variants in several genes, including *ppsC*, *eccD5,* and *eccA5* involved in metabolism and cell wall processes and reportedly associated with virulence in mycobacteria. However, such polymorphisms did not alter the genomic stability and viability of BCG-1 (Russia)-derived vaccines in vivo. Sequence variants occurred in polymorphic CDS of seed lot 361 used for vaccine manufacture before 2006 appeared accumulated in two of six progeny isolates, hypothetically, under selective pressure in the human organism. Although polymorphisms were identified in two unrelated clinical isolates 2925 and 5448 from children immunized in different years with different vaccine lots produced from the BCG-1 (Russia) seed lot 361 by the same manufacturer, the impact of such changes in the context of association with the development of BCG-induced disease remains questionable due to the lack of detailed information on the immune status and host genetics of presumably immunocompetent patients with BCG-osteitis.

Despite promising results in the development of a new generation of BCG vaccines, there is no reliable alternative to those which are still in use in the nearest future.

The contributions of genomic diversity and variations in gene expression profiles during the BCG vaccine manufacture flow and adaptations in vivo are yet to be uncovered in parallel with the molecular mechanisms of innate and adaptive immune responses in the human organism. Thereby, the comparative studies of genomic variations in production strains, their seeds, administered vaccine lots, and the descendant clinical isolates (if available) represent a beneficial approach to better understand the bases of vaccine efficacy and adverse reactions of present and future BCG-based vaccines at genomic, transcriptomic, and proteomic levels.

## Methods

### Study design

We retrospectively reviewed 65 medical records at the clinics of St. Petersburg Research Institute of Phthisiopulmonology selected as codes A18.0 (Tuberculosis of bones and joints, acute or chronic osteomyelitis) and A18.2 (Tuberculosis peripheral lymphadenopathy) according to the International Statistical Classification of Diseases and Related Health Problems (ICD-10). The probable BCG-ID was suspected in nine immunocompetent children (eight patients aged under 5 years and the 14-year old patient) with developed osteitis (eight patients), or cutaneous lesion near the BCG inoculation site (one patient), well-defined BCG inoculation records, and without a history of TB contacts admitted for treatment (including surgical intervention) in 2006–2018 (Table 2).

### Inclusion criteria

The definition for the diagnosis of BCG-ID (“BCG-itis”) included 1) the histology showing granulation tissue with caseous necrosis in biopsy samples taken while surgery and 2) mycobacteria cultures recovered from tissue samples on the Löwenstein-Jensen (L-J) medium if they were identified as *M. bovis* BCG using GenoType MTBC (Hain Lifescience, Germany). The *M. bovis* BCG cultures were stored at − 80 °C for further examination.

### Drug susceptibility testing

Drug susceptibility to first-line antibiotics (streptomycin, isoniazid, rifampin, ethambutol) and pyrazinamide of the *M. bovis* BCG cultures was assessed by using conventional absolute concentration method and/or BACTEC MGIT 960 System (Becton-Dickinson, Sparks, MD, USA).

### BCG-1 (Russia) seed lots and clinical isolates used for WGS

For genomic analysis, we used genomic DNA extracted as described [44] from *M. bovis* BCG-1 (Russia) freeze-dried seed lots and nine BCG clinical isolates cultured on L-J medium, each recovered from a child patient with confirmed BCG-ID.

The lyophilized seed lots of three generations 361 (“sh”), 367 (“shch”), and 368 (“shch”) of *M. bovis* BCG-1 (Russia) substrain No. 700001 in ampoules were obtained from the National State Collection of Pathogenic Microorganisms (NSCPM) of Scientific Center for Expertise of Medical Application Products of the Ministry of Health, Russia along with the characteristics (the origin and manufacture) of corresponding BCG vaccine commercial lots used for immunization (Table 3).

Clinical isolates 1986, 2925, 4159, 5340, 5075, and 5448 - progenies of BCG-1 (Russia) seed lot 361 (“sh”), isolate 3363 – of seed lot 367 (“shch”) and isolate 1032 – of seed lot 368 (“shch”) were obtained from biopsies of patients with BCG-osteitis affecting femur, tibia, calcaneus or ribs; isolate 577 – the progeny of seed lot 368 (“shch”) was obtained from a patient with an abscess at the point of injection (Tables 2 and 3).

### Library construction and DNA sequencing bioinformatics analysis

DNA samples were purified by RNase A (Thermo Scientific™ #EN0531) and sonicated using Bioruptor® UCD-200 system (Diagenode, Denville, NJ, USA) to prepare paired-end (P.E.) libraries by following NEB Library Preparation Protocol, as seen in the product manual for the NEBNext Ultra II DNA Library Preparation Kit for Illumina (NEB #E7645). P.E. genomic libraries were subjected to WGS performed using the MiSeq platform (Illumina, San Diego, CA, USA). The average coverage of 28x was achieved. Raw PE reads were qualitatively analyzed using FastQC software (https://www.bioinformatics.babraham.ac.uk/projects/fastqc/) to retrieve proper parameters for trimming and filtering with Trimmomatic software version 0.32 [45]. Trimmed reads were aligned to the initially indexed reference genome of *M. bovis* BCG-1 (Russia), available at NCBI GenBank under the accession number CP013741 (RefSeq: NZ_CP013741.1) using BWA-MEM algorithm [46]. Sequence alignment maps were subsequently sorted by coordinates with conversion to binary alignment maps, deduplicated and indexed using Picard software version 2.17.8 (http://broadinstitute.github.io/picard/) SortSam, MarkDuplicates, and BuildBamIndex tools, respectively. Next, deduplicated reads were subjected to the realignment based on the realignment target list performed with the Genome Analysis Toolkit (GATK) software version 4.1.1.0 IndelRealigner tool [47]. Realigned reads were used for variant identification using the GATK version 4.1.1.0 HaplotypeCaller tool. Identified variants were selected to generate separate SNPs- and indels-containing variant call format (VCF) files using the GATK version 4.1.1.0 SelectVariants tool. SNPs and indels then were processed with the GATK version 4.1.1.0 VariantFiltration tool according to the following parameters such that SNPs with the quality per depth (Q.D.) less than 2.0, Fisher strand bias (F.S.) more than 60.0, strand odds ratio (SOR) more than 4.0, root mean square mapping quality (M.Q.) less than 40.0, mapping quality rank-sum test (MQRankSum) less than − 12.5, read position rank-sum test (ReadPosRankSum) less than − 8.0 and indels with Q.D. < 2.0, F.S. > 200.0, ReadPosRankSum < − 20.0, SOR > 10.0 were filtered out. Next, realigned reads were subjected to the base quality score recalibration (BQSR) stage using the GATK version 4.1.1.0 BaseRecalibrator and PrintReads tools to model systematic sequencing technical errors, adjust base quality scores accordingly basing on filtered VCF files and generate recalibrated reads, which improves the accuracy of variant calls. Recalibrated reads were used for adjusted variant identification using the GATK version 4.1.1.0 HaplotypeCaller tool. Identified variants were selected to generate separate SNPs- and indels-containing variant call format (VCF) files using the GATK version 4.1.1.0 SelectVariants tool. SNPs and indels were then processed with the GATK version 4.1.1.0 VariantFiltration tool, as described above. Effects on genes and putative impact (modifier, low, moderate, high) of identified variants were predicted using SnpEff software version 4.3 T [48] basing on annotations retrieved from general feature format version 3 (GFF3) file of *M. bovis* BCG-1 (Russia) current seed lot 368 (“shch”) available at NCBI under the accession number CP013741. Particular genome features were retrieved from the PATRIC platform 3.6.2 (https://www.patricbrc.org/view/Taxonomy/1763) [49], UniProt (https://www.uniprot.org/) [50], and MycoBrowser portal (https://mycobrowser.epfl.ch) [51].

The *pncA* gene was searched for mutations conferring resistance to pyrazinamide in BCG clinical isolates [38].

### Comparative genomic analysis

Reference genomic sequences of *M. tuberculosis* H37Rv (NC_000962.3) and *M. bovis* BCG strains: BCG-1 (Russia) (NZ_CP013741; NZ_CP011455; NZ_CP009243), Tokyo-172 type I (NC_012207), Pasteur 1173P2 (NC_008769), BCG Russia (NZ_CUWO01000001), and Danish 1331 (NZ_CP039850) were used to analyze variations in coding DNA sequences (CDS) by the NCBI basic local alignment and search tool (BLAST) (https://blast.ncbi.nlm.nih.gov/Blast.cgi)

## Supplementary information

**Additional file 1: Table S1.** Sequence variants in BCG-1 (Russia) parent seed lots 361, 367, and 368 and their progeny clinical isolates (complete information). Sequence variants are presented as reads with alternative allele/all reads, ratio, %; del, deletion; fs, frameshift.

**Additional file 2: Figure S1.** Comparative circular genome diagram of *M. bovis* BCG 1 (Russia) vaccine seeds and clinical isolates (circos plot was created with Circa (http://omgenomics.com/circa). Tracks from inside: scale in megabases; G.C. content histogram (yellow fill with black stroke) calculated from the ratio (G + C)/(A + T + G + C) using a 1 kilobase (kb) non-overlapping sliding window; G.C. bias plots calculated from the ratio (G C)/(G + C) using a 1 kb non-overlapping window (blue plot) and a 10 kb non-overlapping window (translucent black plot); strain number labeled tracks (alternating grey and white) each depicting strain-specific insertions/deletions (blue/red transverse marks) and SNPs (dots*) identified relative to the reference genome of *M. bovis* BCG-1 (Russia) (GenBank accession number CP013741); tracks depicting ORFs (blue transverse marks) alongside affected CDSs (black, orange and red transverse marks^†^) on forward and reverse strands; names of affected genes colored according to an impact type of the most severe variant affecting a particular gene. Notes. *Sizes and colors determine a particular SNP type: small black – synonymous SNP, large orange – missense SNP; ^†^color determines an impact type of a particular variant on a particular CDS assigned according to the following concept: low impact (black mark) – synonymous SNPs, moderate impact (orange mark) – missense SNPs and conservative insertions/deletions, high impact (red mark) – nonsense SNPs and frameshift insertions/deletions. (PPTX 311 kb)

**Additional file 3: Figure S2.** UspA domain 2: sequence alteration and truncation. (A) Sequences of affected *UspA* gene region. The top two rows depict reference region aligned against respective loci of strain 1032, indicating cytosine deletion in the codon 210. The third row represents the consensus sequence of the region highlighting premature opal (TGA) stop codon. (B) UspA amino acid sequence alteration and protein truncation. Domain 2 altered region and corresponding changed residues indicated in red. Ligand-binding sites depicted as green regions. (PPTX 59 kb)

## Data Availability

Raw sequencing data of BCG-1 (Russia) seed lots 361, 367, and 368 (accession numbers: SRR9593176, SRR9593167, SRR9593166), and nine BCG clinical isolates (accession numbers: SRR9593177, SRR9593172, SRR9593171, SRR9593170, SRR9593169, SRR9593168, SRR9593175, SRR9593174, SRR9593173) are available in the NCBI Sequence Read Archive (https://www.ncbi.nlm.nih.gov/sra) related to the BioProject “Whole-genome sequence variation of *Mycobacterium bovis* BCG-I (Russia) vaccine substrain” (Accession: PRJNA523499). Reference genomic sequences of *M. tuberculosis* H37Rv (NC_000962.3) and *M. bovis* BCG strains BCG-1 (Russia) (CP013741, NZ_CP013741; NZ_CP011455; NZ_CP009243), Tokyo-172 type I (NC_012207), Pasteur 1173P2 (NC_008769), BCG Russia (NZ_CUWO01000001), and Danish strain 1331 (NZ_CP039850) are available at GenBank NCBI (https://www.ncbi.nlm.nih.gov/genbank/). All data generated or analyzed during this study are included in this published article, in particular, in the Methods section (subsection Comparative genomic analysis) and Tables 2 and 3.

## Abbreviations

BCG: Bacille Calmette-Guérin
TB: Tuberculosis
SNP: Single nucleotide polymorphisms
BCG-ID: BCG-induced disease
WGS: Whole-genome sequencing
CDS: Coding DNA sequences
