# Supplementary material for: First insight into the whole-genome sequence variations in Mycobacterium bovis BCG-1 (Russia) vaccine seed lots and their progeny clinical isolates from children with BCG-induced adverse events
Source: BMC Genomics. 2020 Aug 18;21:567. doi: 10.1186/s12864-020-06973-5 (PMC7437937; doi:10.1186/s12864-020-06973-5)
Supplement: Supplementary file 2 — Additional file 2: Figure S1. Comparative circular genome diagram of M. bovis BCG 1 (Russia) vaccine seeds and clinical isolates (circos plot was created with Circa (http://omgenomics.com/circa). Tracks from inside: scale in megabases; G.C. content histogram (yellow fill with black stroke) calculated from the ratio (G + C)/(A + T + G + C) using a 1 kilobase (kb) non-overlapping sliding window; G.C. bias plots calculated from the ratio (G C)/(G + C) using a 1 kb non-overlapping window (blue plot) and a 10 kb non-overlapping window (translucent black plot); strain number labeled tracks (alternating grey and white) each depicting strain-specific insertions/deletions (blue/red transverse marks) and SNPs (dots*) identified relative to the reference genome of M. bovis BCG-1 (Russia) (GenBank accession number CP013741); tracks depicting ORFs (blue transverse marks) alongside affected CDSs (black, orange and red transverse marks†) on forward and reverse strands; names of affected genes colored according to an impact type of the most severe variant affecting a particular gene. Notes. *Sizes and colors determine a particular SNP type: small black – synonymous SNP, large orange – missense SNP; †color determines an impact type of a particular variant on a particular CDS assigned according to the following concept: low impact (black mark) – synonymous SNPs, moderate impact (orange mark) – missense SNPs and conservative insertions/deletions, high impact (red mark) – nonsense SNPs and frameshift insertions/deletions. (PPTX 311 kb) [file 12864_2020_6973_MOESM2_ESM.pptx]

## Slide 1
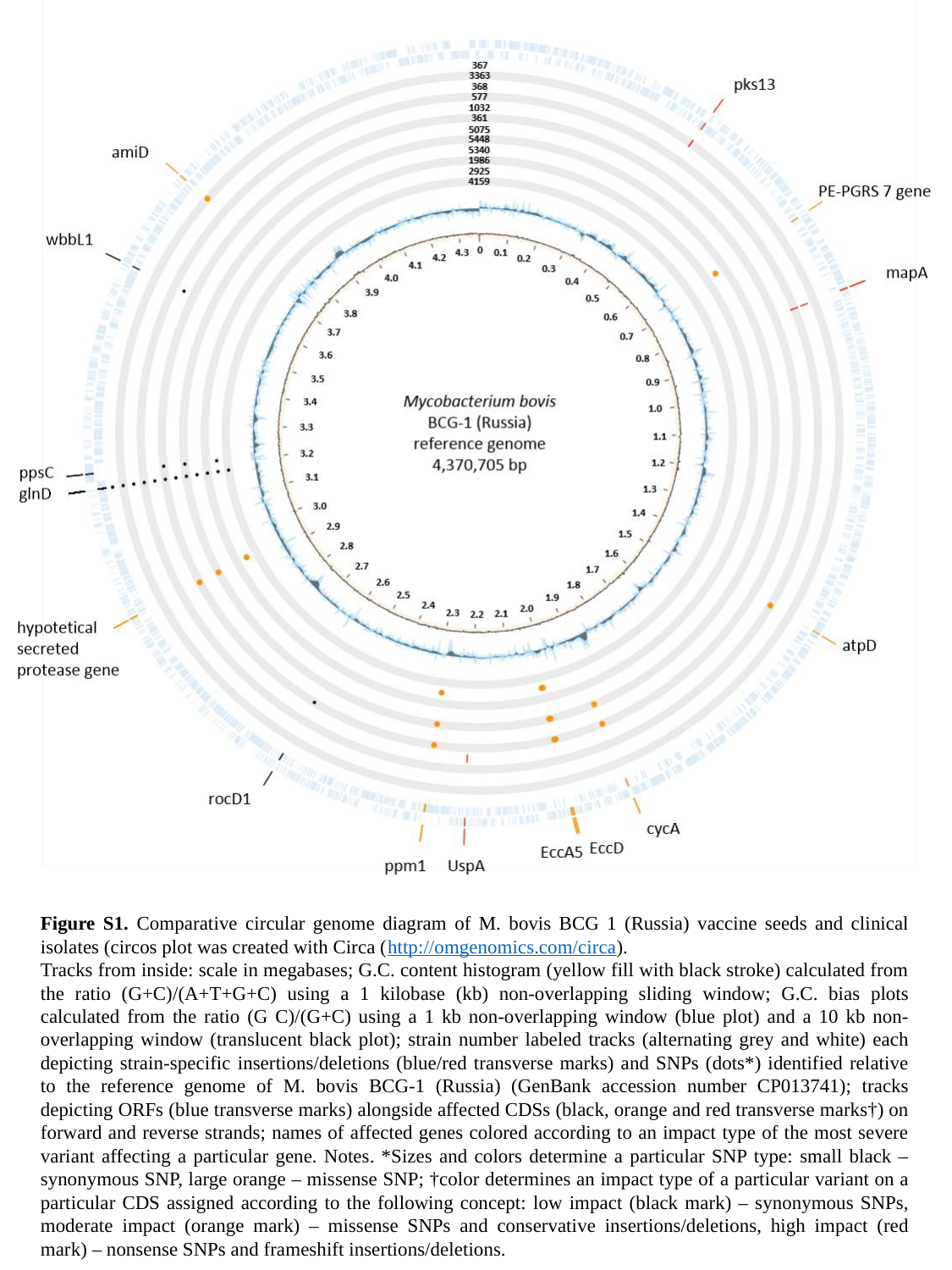

Figure S1. Comparative circular genome diagram of M. bovis BCG 1 (Russia) vaccine seeds and clinical isolates (circos plot was created with Circa (http://omgenomics.com/circa).
Tracks from inside: scale in megabases; G.C. content histogram (yellow fill with black stroke) calculated from the ratio (G+C)/(A+T+G+C) using a 1 kilobase (kb) non-overlapping sliding window; G.C. bias plots calculated from the ratio (G C)/(G+C) using a 1 kb non-overlapping window (blue plot) and a 10 kb non-overlapping window (translucent black plot); strain number labeled tracks (alternating grey and white) each depicting strain-specific insertions/deletions (blue/red transverse marks) and SNPs (dots*) identified relative to the reference genome of M. bovis BCG-1 (Russia) (GenBank accession number CP013741); tracks depicting ORFs (blue transverse marks) alongside affected CDSs (black, orange and red transverse marks†) on forward and reverse strands; names of affected genes colored according to an impact type of the most severe variant affecting a particular gene. Notes. *Sizes and colors determine a particular SNP type: small black – synonymous SNP, large orange – missense SNP; †color determines an impact type of a particular variant on a particular CDS assigned according to the following concept: low impact (black mark) – synonymous SNPs, moderate impact (orange mark) – missense SNPs and conservative insertions/deletions, high impact (red mark) – nonsense SNPs and frameshift insertions/deletions.
