# Supplementary material for: First insight into the whole-genome sequence variations in Mycobacterium bovis BCG-1 (Russia) vaccine seed lots and their progeny clinical isolates from children with BCG-induced adverse events
Source: BMC Genomics. 2020 Aug 18;21:567. doi: 10.1186/s12864-020-06973-5 (PMC7437937; doi:10.1186/s12864-020-06973-5)
Supplement: Supplementary file 3 — Additional file 3: Figure S2. UspA domain 2: sequence alteration and truncation. (A) Sequences of affected UspA gene region. The top two rows depict reference region aligned against respective loci of strain 1032, indicating cytosine deletion in the codon 210. The third row represents the consensus sequence of the region highlighting premature opal (TGA) stop codon. (B) UspA amino acid sequence alteration and protein truncation. Domain 2 altered region and corresponding changed residues indicated in red. Ligand-binding sites depicted as green regions. (PPTX 59 kb) [file 12864_2020_6973_MOESM3_ESM.pptx]

## Slide 1
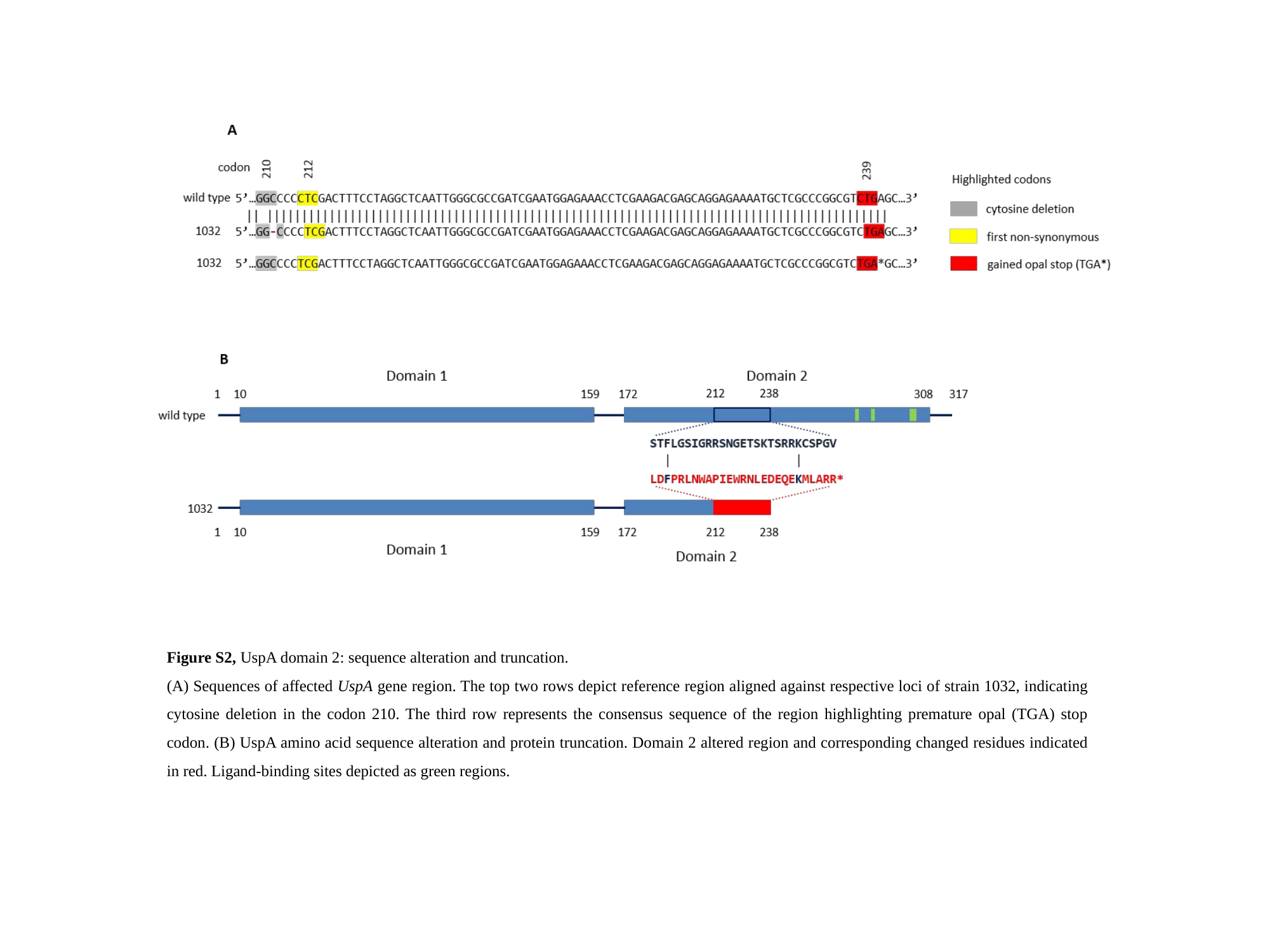

Figure S2, UspA domain 2: sequence alteration and truncation.
(A) Sequences of affected UspA gene region. The top two rows depict reference region aligned against respective loci of strain 1032, indicating cytosine deletion in the codon 210. The third row represents the consensus sequence of the region highlighting premature opal (TGA) stop codon. (B) UspA amino acid sequence alteration and protein truncation. Domain 2 altered region and corresponding changed residues indicated in red. Ligand-binding sites depicted as green regions.
